# Regulatory role of Mss11 in *Candida glabrata* virulence: adhesion and biofilm formation

**DOI:** 10.3389/fcimb.2023.1321094

**Published:** 2024-01-04

**Authors:** Lu-Ling Wang, Si-Jia Huang, Jun-Tao Zhao, Jin-Yan Liu, Ming-Jie Xiang

**Affiliations:** ^1^ Department of Laboratory Medicine, Ruijin Hospital, Shanghai Jiao Tong University School of Medicine, Shanghai, China; ^2^ Department of Laboratory Medicine, Ruijin Hospital Luwan Branch, Shanghai Jiao Tong University School of Medicine, Shanghai, China

**Keywords:** Mss11, *Candida glabrata*, adhesion, biofilm, virulence, EPA, subtelomeric silencing

## Abstract

**Introduction:**

*Candida glabrata* has emerged as a fungal pathogen with high infection and mortality rates, and its primary virulence factors are related to adhesion and biofilm formation. These virulence factors in *C.glabrata* are primarily mediated by epithelial adhesins (Epas), most of which are encoded in subtelomeric regions and regulated by subtelomeric silencing mechanisms. The transcription factor Mss11, known for its regulatory role in adhesion, biofilm formation, and filamentous growth in *Saccharomyces cerevisiae* and *Candida albicans*, has also been implicated in the expression of *EPA6*, suggesting its potential influence on *C.glabrata* virulence. The present study aims to determine the regulatory role of Mss11 in the virulence of *C. glabrata*.

**Methods:**

In this work, a *Δmss11* null mutant and its complemented strain were constructed from a *C.glabrata* standard strain. The impact of the transcription factor Mss11 on the virulence of *C.glabrata* was investigated through a series of phenotypic experiments, including the microbial adhesion to hydrocarbons (MATH) test, adherence assay, biofilm assay, scanning electron microscopy and *Galleria mellonella* virulence assay. Furthermore, transcriptome sequencing, quantitative reverse transcription polymerase chain reaction (RT-qPCR), and chromatin immunoprecipitation sequencing (ChIP-seq) were employed to investigate the molecular mechanisms behind the regulation of Mss11.

**Results:**

In *C.glabrata*, the loss of *MSS11* led to a significant reduction in several virulence factors including cell surface hydrophobicity, epithelial cell adhesion, and biofilm formation. These observations were consistent with the decreased virulence of the *Δmss11* mutant observed in the *Galleria mellonella* infection model. Further exploration demonstrated that Mss11 modulates *C. glabrata* virulence by regulating *EPA1* and *EPA6* expression. It binds to the upstream regions of *EPA1* and *EPA6*, as well as the promoter regions of the subtelomeric silencing-related genes *SIR4*, *RIF1*, and *RAP1*, indicating the dual regulatory role of Mss11.

**Conclusion:**

Mss11 plays a crucial role in *C. glabrata* adhesion and biofilm formation, and thus has a broad influence on virulence. This regulation is achieved by regulating the expression of *EPA1* and *EPA6* through both promoter-specific regulation and subtelomeric silencing.

## 1 Introduction

*Candida glabrata* is an opportunistic pathogenic fungus, which frequently leads to human mucosal, bloodstream, and medical device–associated infections. In recent years, the number of immunocompromised individuals and clinical application of invasive and antibiotic treatments have increased; this has led to a shift in the distribution of invasive infection–causing *Candida* spp. Annually, the isolation rate of *Candida albicans* has been declining, whereas the detection rate of *C. glabrata* has been increasing, with notable geographical variations (Mccarty et al., 2021). For instance, in Australia, the United States, and Malaysia, *C. glabrata* is the predominant candidemia-causing pathogen among the non-albicans *Candida* spp. (Cleveland et al., 2012; Chapman et al., 2017; Farooq et al., 2022); however, in Japan and Europe, it ranks third, with *Candida parapsilosis* frequently detected as the leading or secondary species (Faria-Ramos et al., 2014; Morii et al., 2014). Moreover, compared with those caused by non-albicans *Candida* spp., *C. glabrata* infections are associated with a higher mortality rate, ranging from 40% to 70% (Krcmery and Barnes, 2002).

*C. glabrata* exhibits unique pathogenic characteristics compared with other *Candida* spp. It lacks the ability to form pseudohyphae structures and produce secretory proteases, which are typical *Candida* traits. Consequently, the primary virulence factors of *C. glabrata* are adhesion and biofilm formation (Vale-Silva and Sanglard, 2015). Adhesion to various biotic and abiotic surfaces is the main prerequisite for *C. glabrata* infection, which is primarily mediated by Epa adhesin family proteins, located on the cell wall surface (Silva et al., 2012; Essen et al., 2020). After initial colonization through adhesion, the adhered cells proliferate on the surface, producing extracellular matrix and eventually leading to biofilm formation (Paulitsch et al., 2009). In contrast to the high-density, pseudohyphal-rich biofilms of *C. albicans*, *C. glabrata* biofilms are sparser, being primarily composed of one or many layers of yeast cells with abundant extracellular matrix (Hassan et al., 2021). While the biofilm structure of *C. glabrata* seems relatively benign, it is noteworthy that clinical isolates of *C. glabrata* exhibit the highest biofilm formation rate among all non-albicans *Candida* spp. (Muadcheingka and Tantivitayakul, 2015). Most importantly, biofilm formation is strongly associated with human infections, particularly in *Candida* bloodstream infection cases where 70% of mortality is linked to biofilm formation (Atiencia-Carrera et al., 2022). Given the central role these virulence factors play in *C. glabrata* pathogenicity, understanding the underlying biological mechanisms is imperative.

Mss11, a transcription factor initially identified in *Saccharomyces cerevisiae* for its role in starch degradation, has been the focus of extensive research (Webber et al., 1997). For instance, in *S. cerevisiae*, Mss11 mediates cell adhesion, pseudohypha formation, and invasive growth by regulating the expression of genes encoding cell wall-associated proteins, such as *FLO1*, *FLO10*, and *FLO11* (Gagiano et al., 1999; Bester et al., 2012; Shapiro et al., 2012; Taylor and Ehrenreich, 2014; Bernardi et al., 2018). In *C. albicans*, the influence of Mss11 extends to the control of hyphal development and biofilm formation (Su et al., 2009; Shapiro et al., 2012; Tsai et al., 2014). Moreover, a study on the changes in *C. glabrata* virulence after exposure to chemical antifungal preservatives found the transcriptional activation of *EPA6*, an epithelial adhesin (Epa)-encoding gene in *C. glabrata*, is dependent on Mss11 (Mundy and Cormack, 2009). Thus, Mss11 may have a strong association with yeast virulence. However, the role of Mss11 in *C. glabrata* virulence development remains unknown.

To elucidate the role of Mss11 in *C. glabrata*, we constructed an *Δmss11* null mutant strain from the *C. glabrata* standard strain ATCC 2001 using homologous recombination. Subsequently, the *MSS11* complementation strain was generated using plasmid-based complementation based on the null strain. Our results indicated that Mss11 is vital for the adhesion and biofilm formation abilities of *C. glabrata*, and it consequently impacts its virulence. This modulation is achieved by regulating *C. glabrata* Epa1 and Epa6 expression, which might be mediated through classic promoter-specific regulation and subtelomeric silencing.

## 2 Materials and methods

### 2.1 Strains, media, and culture conditions

**Table 1** lists *C. glabrata* strains and plasmids used in this study. All strains were cultured at 30°C in yeast–peptone–dextrose (YPD) medium consisting of 1% yeast extract, 2% peptone, and 2% glucose, either in YPD broth or on YPD agar (YPD broth supplemented with 1.5% agar). YPD agar plates supplemented with 100 μg/mL nourseothricin and 1000 μg/mL hygromycin were used for NAT- and hygR-resistant strain selection, respectively.

**Table 1:** Strains and plasmids used in this study.

Strain/plasmid	Genotype/description	Source/reference
ATCC 2001	*Candida glabrata* ATCC 2001 (CBS138) strain	ATCC
pYC44	The empty backbone for yeasts, *NAT*	Addgene
*Δmss11*	ATCC 2001 (*Δmss11::NAT*)	This study
*Δmss11*+*MSS11*	ATCC 2001 (*Δmss11::NAT*,pCN-HygR-MSS11)	This study
pCN-PDC1-GFP	The integrating vector of *C. glabrata*, *NAT*, *GFP*	Addgene
pCN-HygR-MSS11	pCN-PDC1-GFP (*Δnat::HygR*, *ΔPDC1::Mss11*)	This study
pCN-PDC1-MSS11-3XFLAG	pCN-PDC1-GFP (*Δnat::HygR*, *Δgfp::Mss11-3XFLAG*)	This study
pCN-PDC1-MSS11	pCN-PDC1-GFP (*Δnat::HygR*, *Δgfp::Mss11*)	This study

To construct the *Δmss11* null mutant strain, the homologous recombination technique of Zhao et al. (Zhao et al., 2022) was employed. In brief, by using the ATCC 2001 strain DNA as the template, the upstream and downstream regions of *MSS11* was amplified with primers MSS11-UP and MSS11-DOWN. Similarly, the NAT resistance marker was amplified using the plasmid pYC44 (Yáñez-Carrillo et al., 2015) and the MSS11-NAT primers (**Table 2**, with reverse complement sequences denoted in lowercase). After fusion polymerase chain reaction (PCR), the knockout cassette was transformed into the ATCC 2001 strain by using the LiAc/single-stranded (SS) carrier DNA/polyethylene glycol (PEG) transformation method to replace the target gene with the selection marker. Subsequently, the transformants that could grow on YPD agar plates containing 100 μg/mL nourseothricin were identified as the *Δmss11* mutants, with further confirmation through PCR with MSS11-Y primers and quantitative reverse transcription PCR (RT-qPCR) with RT-MSS11 primers. To construct a reintegration strain, the plasmid pCN-HygR-MSS11, harboring full-length *MSS11* with its original promoter and a hygromycin resistance marker, was generated from pCN-PDC1-GFP (Zordan et al., 2013) by employing the in-fusion cloning kit (Takara, Shiga, Japan). After the transformation of pCN-HygR-MSS11, colonies that could grow on YPD agar plates containing 1000 μg/mL hygromycin and exhibited comparable *MSS11* expression levels to ATCC 2001 through RT-qPCR were identified as complemented strains. To ensure consistent expression of *MSS11* throughout the experiments, YPD agar plates and YPD broth supplemented with 1000 μg/mL hygromycin were used for routine maintenance and cultivation of the complemented strain, respectively, as described by Zordan et al. (Zordan et al., 2013).

**Table 2:** Primers used in this study.

Primer name	Sequence (5′→3′)
MSS11-UP-F	CAAAAGCATGTGATGGTGTCAA
MSS11-UP-R	cccggacagccgctaggaggtTGGTACGGACATCGGAAGTA
MSS11-DOWN-F	ccgtagcccgatagtcccgagGCGGCTACCTCAGAAACCTC
MSS11-DOWN-R	CAGCAGGCAAACCTCTCTCT
MSS11-NAT-F	acctcctagcggctgtccgggGTTGTAAAACGACGGCCAGT
MSS11-NAT-R	ctcgggactatcgggctacggAGGAAACAGCTATGACCATG
MSS11-Y-F	TCGGGATAATTGCTATGCCC
MSS11-Y-R	TGGCGGTATGGGAAAGGAAC
RT-ACT1-F	TTCCAGCCTTCTACGTTTCC
RT-ACT1-R	TCTACCAGCAAGGTCGATTC
RT-MSS11-F	TGCCTTTCCAACCTTACCCC
RT-MSS11-R	GCGAAGCCCAACACAGAATG
RT-EPA1-F	ACCGCAAGAAAATCCTCCTCC
RT-EPA1-R	TGGTGCTGATGATATTGATTTGTTG
RT-EPA6-F	GAAATCAGGATCGAATCCATG
RT-EPA6-R	GTGGTAATGTATCAAACAGCG
RT-SIR4-F	CAAGGACTCTGGATCGGCAA
RT-SIR4-R	TTCGAGTGCCCACCTTTACC
RT-RIF1-F	TACACCAATCTCAGCAGCCC
RT-RIF1-R	ATTTGCCTTGGCGCGATTTT
RT-RAP1-F	TCGGGCTCGTCATTGTTAGC
RT-RAP1-R	GCGTGTAGCCAAAAGGCAAA

For chromatin immunoprecipitation (ChIP) sequencing (ChIP-seq) analysis, a strain expressing the FLAG-tagged Mss11, as well as its control strain without the FLAG tag was constructed. The method was identical to that used for complemented strain construction. In brief, by using pCN-PDC1-GFP (Zordan et al., 2013) as the template, pCN-PDC1-MSS11-3XFLAG–carrying full-length *MSS11* with the strong PDC1 promoter and the FLAG tag was generated. Analogously, pCN-PDC1-MSS11 harboring full-length *MSS11* alone with the PDC1 promoter was constructed as the control plasmid. After transforming these two plasmids into the ATCC 2001 strain respectively, strains exhibiting hygromycin resistance were further confirmed for the expression of the FLAG-tagged protein through Western blotting.

### 2.2 Cell surface hydrophobicity determination

Cell surface hydrophobicity (CSH) was determined using the microbial adhesion to hydrocarbons (MATH) test, reported by Zhao et al. (Zhao et al., 2022). Yeast grown overnight was adjusted to 1 × 10^6^ cells/mL by using YPD broth, and 2.5 mL of this suspension was inoculated into individual wells of a 6-well cell culture plate. The plate was incubated at 37°C for 2 h; then, the supernatant was aspirated to remove nonadherent cells. Next, fresh YPD broth was replenished, and the plate was incubated at 37°C for an additional 48 h. After incubation, cells were washed with phosphate-buffered saline (PBS) and adjusted to an optical density at 600 nm (OD600) of 1.0 (denoted as A0). Next, 0.6 mL of n-octane was added to 2.4 mL of the yeast suspension. The two-phase system was vortexed and centrifuged at 8,000 rpm for 5 min, enabling the separation of the aqueous and organic phases. Subsequent to centrifugation, the OD600 of the aqueous phase was immediately determined as A1. The A1 value per experiment was the average of three repeated measurements. The data presented are the average A1 values of three experiments. CSH was calculated as.


$$CSH=\frac{A0-A1}{A0}\times 100\%$$

### 2.3 Adherence assay

The adherence assay was performed as described by Chen et al. (Chen et al., 2023) with some modifications. In brief, the epithelial cell lines 293T and Caco-2 were cultured in Dulbecco’s modified Eagle’s medium (DMEM) containing 10% and 20% fetal bovine serum, respectively, at 37°C with 5% CO_2_. Cells were seeded in 24-well plates at 1 × 10^6^ cells/well in 1 ml of culture medium and allowed to grow to full confluence at 37°C with 5% CO_2_. To prepare *C. glabrata* suspensions for adhesion, overnight cultures were diluted in fresh medium and grown until they reached the mid-log phase. Next, the yeast cells were collected, washed, and adjusted to an OD600 of 0.5 with PBS. After 10-fold dilution with DMEM, the cells were added to 24-well plates and co-incubated with epithelial cells at 37°C with 5% CO_2_ for 1 h. Nonadherent cells were removed by washing, and adherent cells were recovered through lysis with 0.1% Triton X-100 and plated onto YPD agar plates for quantification of colonies. The experiments were repeated three times per sample and three biologically independent clones for each strain were examined.

### 2.4 Biofilm assay

For biofilm formation quantification and metabolic activity measurement, crystal violet staining and XTT reduction assay were used, respectively. For both protocols, a single *C. glabrata* colony was inoculated into YPD broth and then cultured at 30°C with shaking at 200 rpm overnight. The overnight culture was resuspended in fresh YPD and grown at 30°C with shaking for 4h to reach the exponential phase. Then, the culture suspension was adjusted to an OD600 of 1.0.

To quantify biofilm formation, 100 μL of the yeast suspension was added to a 96-well plate, followed by incubation at 30°C for 1–24 h. Next, nonadherent cells were washed off by using sterile PBS, and the biofilm was stained using crystal violet. The results were measured through spectrophotometry at 570 nm. For biofilm metabolic activity measurement, the yeast suspension was adjusted to the final concentration of 3.6×10^3^ cells/mL and then added to a 96-well plate at 100 μL per well. After 24 h of incubation, nonadherent cells were removed using PBS, and 100 µl of a solution containing 0.5 mg/mL XTT and 1 μM menadione was added to each well. After incubation in dark for 2 h, the XTT reduction was measured in terms of absorbance at 490 nm. The experiment was carried out in three biologically independent assays and each sample was tested in triplicate, with sterile YPD broth used as the negative control.

### 2.5 Scanning electron microscopy

Scanning electron microscopy (SEM) was employed to visualize *C. glabrata* biofilm structures. The experiments were repeated three times per sample and at least two independent clones for each strain were examined. To allow for biofilm formation, the overnight culture was collected, washed twice with PBS, and diluted to an OD600 of 0.1 with YPD broth. Then, 2 mL of the cell suspension was added to a 24-well microtiter plate with plastic coverslips (Nunc Thermanox; Thermo Scientific). After incubation at 37°C for 24 h, the plastic coverslips were washed twice with PBS and fixed in 2.5% glutaraldehyde at 4°C overnight. The samples were washed three times with PBS and postfixed with 1% osmium tetroxide solution at 4°C for 1–2 h. Subsequently, the samples were washed three times with PBS and then dehydrated serially in increasing concentrations of ethanol: 30%, 50%, 70%, 80%, and 90% ethanol (each for 15 min); this was followed by submersion in 100% ethanol at 4°C for 20 min three times. The samples were then dried in an EM CPD300 critical point dryer (Leica, Germany), fixed to the specimen stage, and coated with a 10-nm gold layer using JEE-420 sputter coater. Finally, biofilm structures were examined and photographed under a scanning electron microscope (SU8010; Hitachi).

### 2.6 Virulence assay using a *Galleria mellonella* infection model

To investigate the virulence of different *C. glabrata* strains, *Galleria mellonella* was used as the infection model organism. The virulence assay was performed as described by Ames et al. (Ames et al., 2017) with some modifications. In brief, mid-log phase yeast cells were harvested, washed, and resuspended to a density of 1 × 10^8^ cells/mL in PBS. To initiate infection, groups of 10 randomly chosen *G. mellonella* larvae, weighing 0.25–0.35 g, were inoculated by injecting 10 µL of the previously prepared yeast cell suspensions into the hemocoel of their last left proleg with a Hamilton syringe. An additional 10 larvae were injected with 10 µL of PBS as a negative control. After infection, the larvae were kept in a humidified incubator at 37°C, and their viability was monitored on the basis of their response to physical stimulation daily. Throughout the experiment, three independent biological replicates were performed, and each sample was examined three times.

### 2.7 RNA extraction and RT-qPCR

The approaches used for RNA extraction and RT-qPCR were performed according to the methods reported by Chen et al. (Chen et al., 2023). In brief, mid-log–phase *C. glabrata* cells were harvested and washed. Total RNA was then extracted using the Yeast RNAiso Kit (Takara), followed by treatment with gDNA Eraser (Takara) to eliminate residual gDNA. The purified RNA was subsequently converted to cDNA by using the PrimeScript RT Reagent Kit (Takara). The mRNA levels of the target genes were measured through quantitative real-time PCR with TB Green Premix Ex Taq (Tli RNaseH Plus, ROX plus) (Takara) on the 7300 Real-Time PCR System (Applied Biosystems, Beijing, China), according to the manufacturer’s instructions. For relative mRNA quantification, the 2^−ΔΔCT^ method was used, with *ACT1* as the internal control. Gene expression was statistically analyzed using GraphPad Prism, with a *P* value of less than 0.05 considered to indicate significance. The data presented in this study originates from three independent experiments, with each sample tested three times. All PCR primers used here are listed in **Table 2**.

### 2.8 Transcriptome sequencing

Transcriptome sequencing was conducted as described by Chen et al. (Chen et al., 2023). First, for library construction, total RNA was extracted from mid-log–phase cells by using Trizol (Invitrogen, Waltham, MA, USA). Next, the mRNA was enriched and fragmented. By using the SuperScript double-stranded cDNA synthesis kit (Invitrogen, CA) with random primers, double-stranded cDNA was reverse-transcribed from the mRNA template. The synthesized cDNA was then modified, selected, and amplified to generate the final library. After quantification using Qubit 4.0, the library was sequenced using the Illumina NovaSeq 6000 sequencer. For further bioinformatics analysis, raw sequencing data were first processed using fastp (https://github.com/OpenGene/fastp), to obtain the high-quality clean data. Next, the clean data were then aligned to the reference genome using HiSat2 (http://ccb.jhu.edu/software/hisat2/index.shtml), resulting in mapped data suitable for subsequent transcriptome assembly and expression quantification. Gene abundance was quantified using RSEM (http://deweylab.github.io/RSEM/) to obtain Read Counts for each gene; then, DESeq2 (http://bioconductor.org/packages/stats/bioc/DESeq2/) was used to identify differentially expressed genes (DEGs) between samples. The criteria for significantly DEG selection were *P* value<0.05 and fold change≥1.2. Furthermore, Gene Ontology (GO) enrichment analysis was performed using GOatools (https://github.com/tanghaibao/GOatools). GO terms were considered significantly enriched when Bonferroni-corrected *P* value was ≤ 0.05 compared with the whole-transcriptome background.

### 2.9 ChIP-seq

For ChIP library construction and sequencing, DNA extracted from mid-log–phase yeast cells was fragmented through sonication. A portion of the fragmented DNA was then reserved as input. ChIP was performed using the GenSeq ChIP Kit (GenSeq Inc.) and the DYKDDDDK Tag antibody (Cell Signalling), according to the manufacturer’s instructions. The ChIPed DNA was quantified using Quant IT fluorescence assay (ThermoFisher) and subsequently processed for library generation using the GenSeq Rapid DNA Library Prep Kit (GenSeq). Library quality was then determined using the Agilent 2100 Bioanalyzer (Agilent), and the constructed library was sequenced on the Illumina NovaSeq sequencer. To analyze the sequencing results further, initial quality control was conducted using Q30. After adaptor trimming and low-quality read removal by using cutadapt (version 1.9.3), high-quality clean reads were generated. These reads were then aligned to the *C. glabrata* reference genome (ASM254v2) using bowtie2 (version 2.2.4). Peak calling was performed with MACS (version 2.2.7.1). Differentially enriched regions between the FLAG-tagged and untagged Mss11 strains were identified using diffReps (version 1.55.4). A threshold of *P* value<0.05 and fold change≥2 was set for differential enrichment. The differentially enriched peaks were visualized using IGV (version 2.16.2). The genomic distribution and proportional diagram of the enriched peaks were plotted using Python.

### 2.10 Statistical analysis

All data are presented as the mean ± SD from multiple experimental repetitions. Statistical analysis and graphical representation were conducted using GraphPad Prism 8 (GraphPad Inc., La Jolla, CA, USA). Kaplan-Meier curves, analyzed through Mantel-Cox log-rank analysis, were employed to demonstrate survival. Comparisons between two groups were performed using Student’s t-test, considering *P*-values lower than 0.05 as significant.

## 3 Results

### 3.1 *MSS11* deletion reduces *C. glabrata* cell surface hydrophobicity

**Figure 1:**
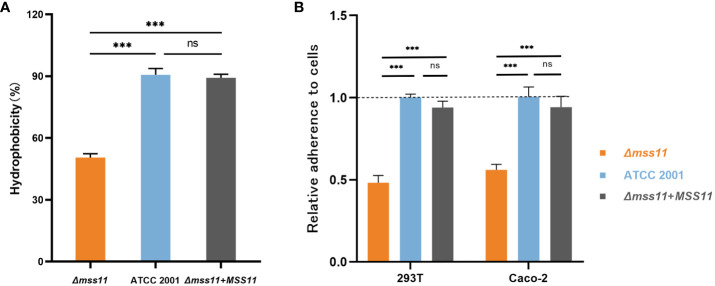
CSH and epithelial cell adhesion of *C. glabrata* ATCC 2001, *Δmss11* mutant, and the complemented strains. **(A)** CSH of ATCC 2001, *Δmss11* mutant, and the complemented strains. The higher the n-octane affinity, the greater is the hydrophobicity. The *Δmss11* mutant exhibited significantly lower CSH than ATCC 2001 and the complemented strains—suggesting a major influence of *MSS11* disruption on CSH in *C. glabrata*. All data are presented as mean ± SD from three independent experiments. **(B)** Adhesion of *C. glabrata* strains to epithelial cells. *C. glabrata* strains were co-incubated with 293T or Caco-2 cells, and the adhesion rate was calculated using the final colony-forming units observed on the plate (relative to ATCC 2001 as 1.0). The adhesion rates of the *Δmss11* mutant to 293T and Caco-2 cells were significantly lower than ATCC 2001 and the complemented strain, suggesting that the absence of *MSS11* in *C. glabrata* significantly impaired its adhesion to epithelial cells. ***P < 0.001, “ns” denotes no significance.

Cell surface hydrophobicity (CSH) is a virulence factor in *C. glabrata*. In this study, the MATH test was used to investigate the relationship between Mss11 and CSH in *C. glabrata*. Yeast cells were vortexed in aqueous and n-octane phases, where the higher the affinity to the n-octane phase, the greater the hydrophobicity. The CSH of the ATCC 2001 strain, *Δmss11* mutant strain, and *Δmss11* mutant’s complemented strains was 90.7%, 50.5%, and 89.2%, respectively. As such, compared with the standard ATCC 2001 strain, the *Δmss11* mutant strain had significantly lower CSH (*P*< 0.001). In contrast, the complemented and ATCC 2001 strains exhibited similar CSH (*P* > 0.05; **Figure 1A**). This suggests that the absence of *MSS11* in *C. glabrata* significantly diminishes its CSH.

### 3.2 Loss of *MSS11* reduces epithelial cell adhesion in *C. glabrata*

Adherence to host surfaces represents the initial step of *Candida* infection. To determine the regulation of Mss11 on *C.glabrata* adhesive properties, we employed two epithelial cell lines, including 293T and Caco-2 cells, to test the adhesion ability of *C. glabrata*. After coincubation of *C. glabrata* and epithelial cells to facilitate adhesion, nonadherent *C. glabrata* cells were washed off. Subsequently, the epithelial cells were lyzed and the associated adherent yeasts were plated. The number of colonies on the plate was considered to represent the epithelial cell adhesion ability of *C. glabrata*. Colony counting results indicated that the adhesion rates of the *Δmss11* null mutant strain to 293T and Caco-2 were 0.48 and 0.56 times that of the ATCC 2001, respectively (both *P*< 0.001). Moreover, the complementation of *MSS11* restored the adhesion rate to an almost similar level as that of the ATCC 2001 (*P* > 0.05; **Figure 1B**). Thus, the absence of *MSS11* significantly reduces epithelial cell adhesion in *C. glabrata*.

### 3.3 *Δmss11* mutant exhibits weakened biofilm formation capability

The role of Mss11 in biofilm formation in *C. glabrata* was investigated by two methods: quantifying biofilm formation using crystal violet staining and evaluating biofilm activity through the XTT assay. Moreover, we used SEM to visually demonstrate the effects of Mss11 on the biofilm morphology of various *C. glabrata* strains.

**Figure 2:**
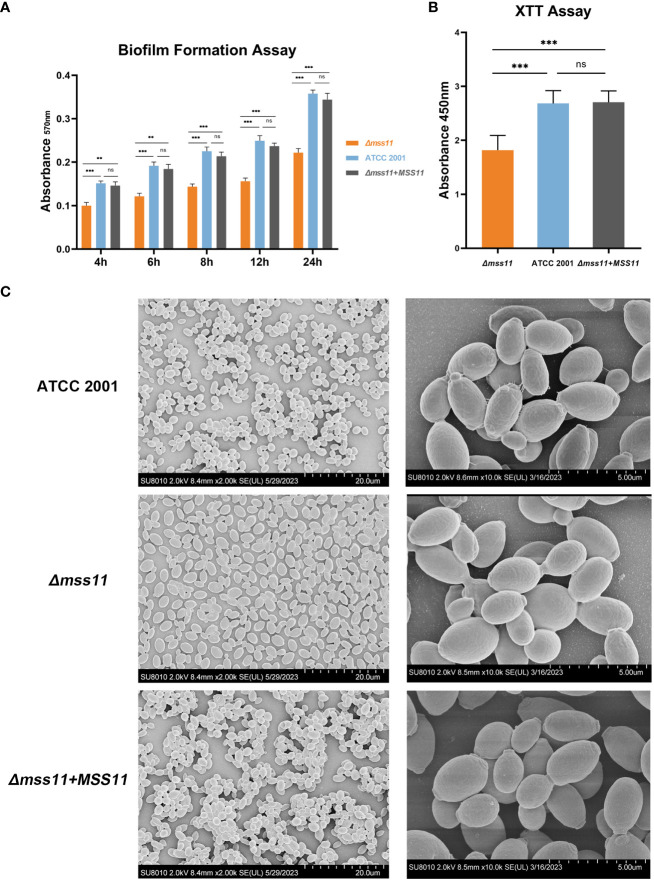
*C. glabrata* biofilm formation analysis. **(A)** *In vitro* biofilm formation assay for quantifying ATCC 2001, *Δmss11* mutant, and the complemented strain biofilms. Absorbance at 570 nm of the mature biofilms was determined after crystal violet staining. The *Δmss11* mutant exhibited significantly lower biofilm biomass than ATCC 2001 and the complemented strains at 4, 6, 8, 12, and 24 (h) **(B)** XTT assay for biofilm viability. Absorbance at 450 nm indicated that the *Δmss11* mutant had significantly lower biofilm metabolic activity than ATCC 2001 and the complemented strains. Results in **(A)** and **(B)** collectively highlight that the absence of *MSS11* not only reduces biofilm biomass but also impairs the biofilm viability significantly in *C. glabrata*. **(C)** SEM of contrasting biofilm structures of ATCC 2001, *Δmss11* mutant, and the complemented strains. Observed variations in cell–cell adhesion (tight to loose arrangement) and extracellular matrix contents (abundant to nearly absent) were consistent with the differences noted in the virulence phenotypes. **P < 0.01, ***P < 0.001, “ns” denotes no significance.

In the crystal violet assay (**Figure 2A**), at 30°C for 4, 6, 8, 12, and 24 h, the total biofilm biomass produced by the *Δmss11* mutant was significantly lower than that produced by the ATCC 2001 and complemented strains (*P*< 0.001), whereas the differences between the ATCC 2001 and complemented strains were nonsignificant (*P* > 0.05). Thus, the absence of Mss11 reduces biofilm formation at various timepoints in *C. glabrata* biofilm lifecycle.

XTT is generally used to measure cell proliferation, but it can also be used for assessing biofilm activity. During coincubation with XTT, mitochondrial dehydrogenases in metabolically active cells within the biofilm reduce XTT to a water-soluble, brown formazan product, the absorbance of which can be detected at 450 nm; moreover, this absorbance value is positively correlated with the metabolic activity of biofilms. Our results revealed that the absence of Mss11 significantly diminished the metabolic activity of mature biofilms in *C. glabrata*. As indicated in **Figure 2B**, the absorbance at 450 nm was 2.68 and 2.7 for ATCC 2001 and the complemented strains, respectively; in contrast, it was only 1.8 for the *Δmss11* mutant. Therefore, the absence of Mss11 not only reduces the total quantity of *C. glabrata* biofilms but also diminishes their metabolic activity significantly.

Our SEM observations of mature biofilm structures of various *C. glabrata* strains (**Figure 2C**) revealed that ATCC 2001 and the complemented strains formed multilayered biofilms with closely arranged cells and evident extracellular matrix. In contrast, the *Δmss11* mutant strain formed the monolayered biofilm, with loosely arranged cells and without apparent intercellular connections or extracellular matrix.

### 3.4 *C. glabrata* requires Mss11 for virulence in *G. mellonella*

**Figure 3:**
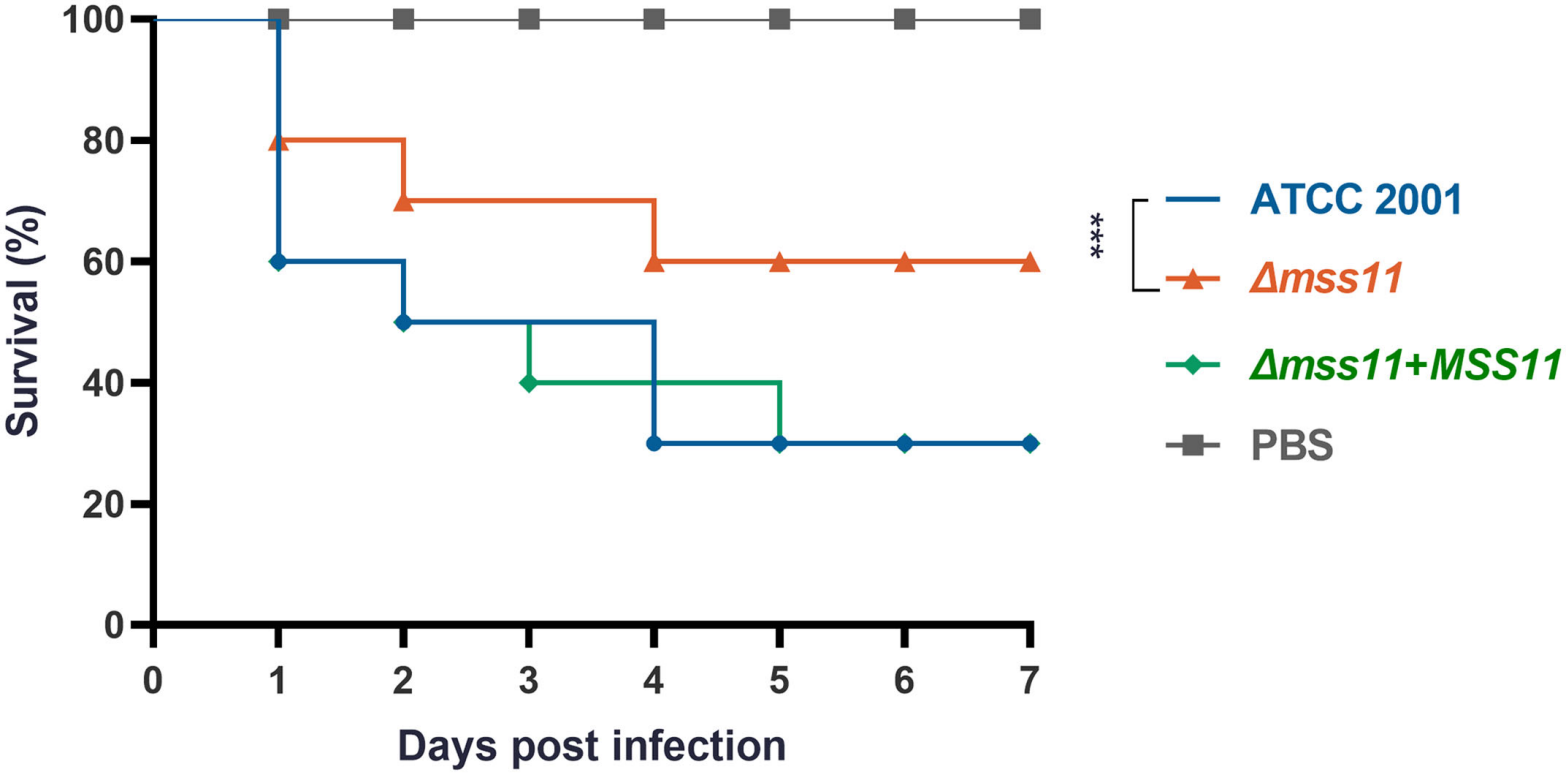
Impact of Mss11 on *C. glabrata* virulence in *G. mellonella* infection model. Healthy larvae were divided into groups and injected with the *C. glabrata* strain suspension or sterile PBS (negative control). Mortality rate of *G. mellonella* larvae was monitored daily based on their response to touch. The virulence of the *Δmss11* mutant was notably diminished, with a 60% survival rate at post-infection day 5; however, it was only 30% for ATCC 2001 and the complemented strains. ***P < 0.001.

Given that Mss11 was noted to have a significant influence on both the adhesive and biofilm formation abilities of *C. glabrata*, we hypothesize that Mss11 also modulates *C. glabrata* virulence. To validate this hypothesis, we used *G. mellonella*, a widely accepted model organism, to establish an *in vivo* infection model. Healthy *G. mellonella* larvae were randomly selected and divided into four groups, injected with either sterile PBS or the suspension of ATCC 2001, *Δmss11* mutant, and the complemented strain. Next, the larvae were monitored daily for vitality; no response to touch was considered the indicator of mortality. Our observations revealed that the *Δmss11* mutant’s virulence was significantly diminished compared with that of both ATCC 2001 and the complemented strains (*P*< 0.001; **Figure 3**). At post-infection day 5, the survival rate of the larvae injected with ATCC 2001 or the complemented strains was 30%, whereas it was 60% for those injected with the *Δmss11* mutant. Therefore, *MSS11* deletion leads to weakened *C. glabrata* virulence toward *G. mellonella*.

### 3.5 Mss11 regulates *EPA1* and *EPA6* expression

*C. glabrata* virulence factors including adhesion and biofilm formation are closely associated with Epa expression. In other words, the types and quantities of Epa can significantly influence the pathogen’s virulence. Through a series of phenotypic assays, we observed that Mss11 plays a regulatory role in the virulence phenotypes of *C. glabrata*. In addition to the relationship found between Mss11 and Epa6, we postulated that Mss11 might modulate virulence factors through Epa regulation.

**Figure 4:**
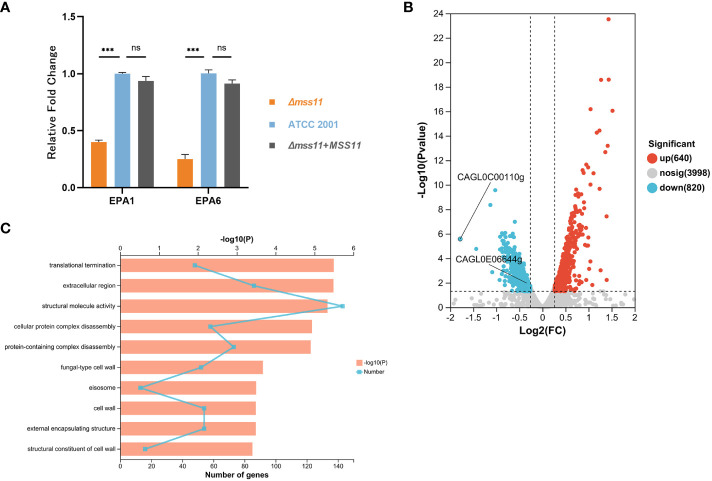
Analysis of *EPA1* and *EPA6* expression in *C. glabrata*. **(A)** RT-qPCR validating *EPA1* and *EPA6* expression levels in various *C. glabrata* strains. A substantial reduction was noted in *EPA1* and *EPA6* expression in the *Δmss11* mutant compared with that in ATCC 2001 and the complemented strains, confirming that Mss11 had a regulatory impact on *EPA1* and *EPA6* expression. **(B)** Transcriptome sequencing for DEGs between ATCC 2001 and *Δmss11* mutant strains. In total, 640 and 820 genes were upregulated and downregulated after *MSS11* deletion, respectively. Notably, *EPA1* (CAGL0E06644g) and *EPA6* (CAGL0C00110g) were among the downregulated genes. **(C)** GO enrichment analysis on DEGs between ATCC 2001 and *Δmss11* mutant strains for Mss11-associated pathways. The identified top 10 enriched pathways were primarily linked to fungal cell wall biosynthesis and extracellular matrix formation. ***P < 0.001, “ns” denotes no significance.

To validate this hypothesis, we first performed transcriptomic sequencing to analyze DEGs between ATCC 2001 and *Δmss11* mutant strains. The results revealed that *MSS11* deletion led to the upregulation of 640 genes and downregulation of 820 genes, including *EPA1* (CAGL0E06644g) and *EPA6* (CAGL0C00110g; **Figure 4B**). Next, we performed RT-qPCR to confirm the relative expression levels of the Epa genes in ATCC 2001, *Δmss11* mutant, and the complemented strains. The results demonstrated that *EPA1* and *EPA6* expression in the *Δmss11* mutant decreased to approximately 0.40 and 0.29 times that of ATCC 2001 (*P*< 0.001), respectively, confirming the regulatory role of Mss11 in *EPA1* and *EPA6* expression (**Figure 4A**). Moreover, our GO enrichment analysis on DEGs between ATCC 2001 and the *Δmss11* mutant strains revealed pathways associated with Mss11. The top 10 significantly enriched pathways were mostly related to fungal cell wall biosynthesis and extracellular matrix formation (**Figure 4C**), consistent with our observations of the correlation among Mss11, cell wall surface proteins, and virulence factors in *C. glabrata*.

### 3.6 Mss11 binds upstream of *EPA1 and EPA6* and promoter of subtelomeric silencing–related genes

**Figure 5:**
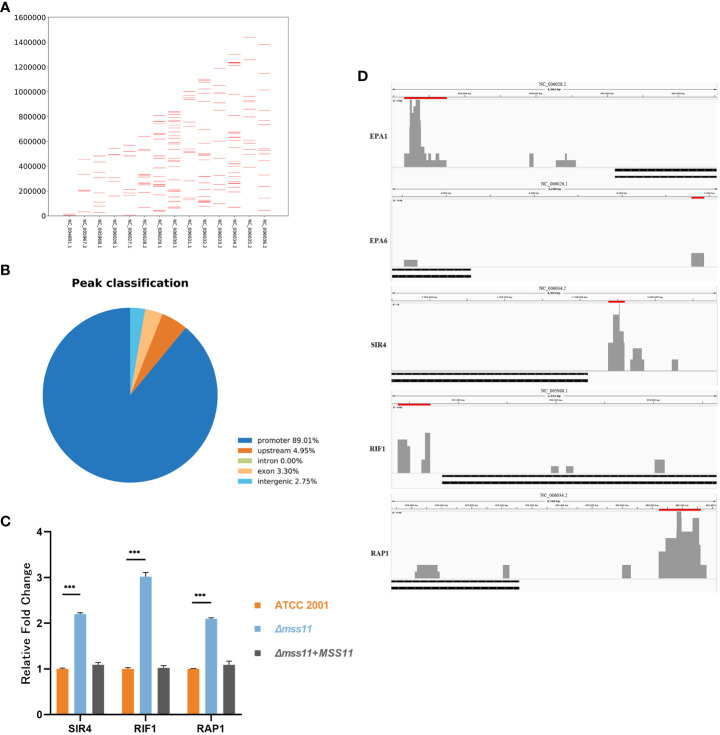
Mss11 binding patterns in *C. glabrata*. **(A)** Comprehensive overview of the binding patterns of Mss11 across the *C. glabrata* genome. **(B)** Pie chart of the distribution of Mss11 DNA binding regions. Most Mss11 binding sites were situated within gene promoter regions, consistent with the expected transcription factor function of Mss11. **(C)** RT-qPCR validating that Mss11 disruption increased expression of subtelomeric silencing-associated genes *SIR4*, *RIF1*, and *RAP1* in *C. glabrata*. ***P < 0.001. **(D)** Differential enrichment peaks in the comparison of ChIP-seq data between FLAG-tagged and untagged Mss11 visualized using IGV. The presented figure is a screenshot from IGV. Red bars above the peaks represent the regions of the differentially enriched peaks and their genomic positions, while black bars below indicate the target genes. The results reveal that Mss11 not only binds to *EPA1* and *EPA6* upstream regions but also to *SIR4*, *RIF1*, and *RAP1* promoters.

To further analyze the regulation of *EPA1* and *EPA6* by Mss11, we constructed the FLAG-tagged Mss11 and conducted ChIP-seq. **Figure 5A** illustrates the overall abiding features of Mss11 across the *C. glabrata* genome. We observed that Mss11 exhibited extensive binding across all chromosomes of *C. glabrata*, with enriched peaks predominantly concentrated within specific genomic features (**Figure 5B**). In particular, most Mss11 binding sites were located within promoter regions of the genes (89.01%), with a smaller fraction in the upstream regions (4.95%) and the remainder distributed among the exon (3.30%) and intergenic (2.75%) regions. This binding pattern aligned with the expected behavior of Mss11 as a transcription factor.

Notably, our investigation revealed that Mss11 binds to approximately 2829 bp upstream of *EPA1* and 2576 bp upstream of *EPA6*, as well as to the promoter regions of the subtelomeric silencing-related genes *SIR4*, *RIF1*, and *RAP1* (**Figure 5D**). Subsequent RT-qPCR also confirmed that in the *Δmss11* mutant, *SIR4*, *RIF1*, and *RAP1* expression levels increased significantly by 2.2-, 3-, and 2.1-fold relative to that in the ATCC 2001, respectively (*P*< 0.001). The expression levels of these genes in ATCC 2001 and the complemented strains were nearly identical (*P* > 0.05; **Figure 5C**), Therefore, Mss11 has a regulatory role in the expression of the subtelomeric silencing–associated genes *SIR4*, *RIF1*, and *RAP1*.

## 4 Discussion

*Candida glabrata*, being devoid of several typical virulence factors found in other *Candida* species, demonstrates a globally increasing trend in clinical prevalence and elevated mortality, emphasizing the importance of delving deeper into its virulence regulatory mechanisms (Alkhalifa et al., 2023; Arendrup et al., 2023; Otto and Babady, 2023). The pathogenicity of *C.glabrata* is primarily attributed to its superior adhesive capabilities and proficiency in biofilm formation (Galocha et al., 2019). The transcription factor Mss11 has been identified in *S. cerevisiae* and *C. albicans* as associated with these virulence factors, leading us to investigate its role in the virulence regulation of *C. glabrata* (Gagiano et al., 2003; Bester et al., 2012; Scherz et al., 2014; Tsai et al., 2014). In the present report, we evaluated its impact on virulence factors, including cell surface hydrophobicity (CSH), cell adhesion and biofilm formation, while elucidating the underlying molecular regulatory mechanisms.

The physicochemical properties of microbial cell surfaces are essential for the growth and metabolism of microorganisms. CSH is one of the most significant physicochemical characteristics of microbial cells, closely associated with nosocomial microbial infections. The correlation of CSH with virulence factors of *Candida* has been investigated extensively. CSH has been noted to be positively correlated with *Candida* adhesion capability to both biotic and abiotic surfaces, as well as *Candida* biofilm formation capacity (Samaranayake et al., 1995; Panagoda et al., 1998; Dabiri et al., 2018). Moreover, given the relative simplicity of CSH detection methods, they can be used for inferring the biofilm formation ability of *Candida* spp. (Borghi et al., 2011). Here, we observed that *MSS11* deletion led to a significant reduction in CSH in *C. glabrata*. Based on these results, we preliminarily hypothesized that Mss11 plays a major role in *C. glabrata* adhesion and biofilm formation.

To elucidate the role of Mss11 in *C. glabrata*’s adherence to biological surfaces and biofilm formation further, we first evaluated the epithelial cell adhesion capacity of various *C. glabrata* strains. Our findings suggested that the absence of *MSS11* diminishes the ability of *C. glabrata* to colonize host epithelial cells. This observation aligns with previous findings in *S. cerevisiae*, where Mss11 was determined associated with cell-cell adhesion and flocculation phenotypes (Bester et al., 2012). Subsequent crystal violet and XTT assays were employed to assess the quantity and viability of *C. glabrata* biofilms. Consistent with the role of Mss11 previously identified in *C. albicans* biofilm formation (Tsai et al., 2014), we observed that *MSS11* loss leads to a significant reduction in both the total amount and activity of *C. glabrata* biofilms; these results were also corroborated by our SEM observations. Moreover, given the critical role of adhesion and biofilm formation in *C. glabrata* virulence, *MSS11* deletion first impacted the virulence factors and was eventually found to be associated with weakened virulence of *C. glabrata* in *G. mellonella*. Notably, several studies have reported the association between *C. glabrata* biofilm formation and antifungal resistance (Rodrigues et al., 2017; Rodríguez-Cerdeira et al., 2019; Gupta et al., 2021), but our research did not observe any significant impact of *MSS11* deletion on antifungal susceptibility (**Supplementary Table 1**).

*C. glabrata* adhesion and biofilm formation are intrinsically associated with its Epas. Our integrative analysis using transcriptome sequencing and RT-qPCR revealed a considerable decrease in the expression of the epithelial adhesin genes *EPA1* and *EPA6* in the *Δmss11* mutant. However, the expression levels of other characterized adhesin genes such as *AWP1*, *AWP2*, *AWP3*, *AWP4*, *AWP5*, *AWP6*, *AWP7*, *AWP12*, *AED1*, *EPA3*, and *EPA22* remained unchanged. *EPA1* and *EPA6* have been found to be associated with various virulence factors in previous studies. Epa1 mediates the adherence to mammalian epithelial cells and phagocytes such as vaginal epithelial cells, macrophages, and monocytes in peripheral blood. It is responsible for approximately 95% of the *in vitro* adhesion ability of *C. glabrata*, and its deletion alone can reduce the adhesion ability to the background levels (Ielasi et al., 2012; Kuhn and Vyas, 2012; Zhao et al., 2022). Epa6 is a major adhesin involved in *C. glabrata* biofilm formation. Iraqui et al. (Iraqui et al., 2005) demonstrated that a yeast lacking Epa6 forms smaller colonies during the biofilm formation process, leading to defective biofilm development. Based on these observations, we proposed a hypothesis that Mss11 modulates *C. glabrata* adhesion and biofilm formation abilities by regulating *EPA1* and *EPA6* expression. However, to further confirm the validity of the aforementioned results, experiments such as generating *MSS11*-knockout, complemented, and overexpression strains in the absence of *EPA1* and *EPA6* are required.

For the transcriptome sequencing data analysis, in addition to the previously demonstrated GO enrichment pathways related to the cell wall and extracellular matrix, we observed enrichment in several other functional GO pathways, such as oxidative reduction (GO:0016491) and intracellular carbohydrate metabolism (GO:0044262; GO:0005975) pathways. The presence of the intracellular carbohydrate metabolism pathway is consistent with findings by Gagiano M et al. (Gagiano et al., 2003), suggesting that Mss11 regulates starch metabolism in *S. cerevisiae*, which indicates the relationship between Mss11 and these phenotypes in *C. glabrata* warrants further exploration. Furthermore, studies have indicated that in both *S. cerevisiae* and *C. albicans*, Mss11 can form a complex with Flo8 to coregulate the expression of the downstream genes. However, whether such a cooperative interaction exists in *C. glabrata* requires further investigations.

In *C. glabrata*, most Epa-encoding genes, including *EPA1* and *EPA6*, are located in subtelomeric regions, and their expression is subject to chromatin-based subtelomeric silencing (De Las Peñas et al., 2003; Castaño et al., 2005). The subtelomeric silencing of Epa-encoding genes in *C. glabrata* principally involves the Sir complex (Sir2, Sir3, and Sir4), Rif1, and Rap1. In some cases, such as *EPA4* and *EPA5* located at the right telomere of chromosome I, as well as *EPA6* and *EPA7* located at both ends of chromosome C, yKu proteins are additionally required for subtelomeric silencing establishment (Rosas-Hernández et al., 2008). The specific mechanisms underlying the function of subtelomeric silencing components have been elucidated in the evolutionarily closely related *S. cerevisiae*. Subtelomeric silencing begins with the binding of Rap1 to telomeric repeat sequences. Subsequently, Rap1 recruits the Sir complex, ultimately leading to the formation of a complex including Sir2, Sir3, and Sir4, collectively mediating the silencing of the corresponding Epa-encoding genes (Rusche et al., 2003). During this process, Rif1 shortens the telomere and yKu proteins elongate it; they jointly maintain telomere length within a normal range to ensure the regular functioning of the subtelomeric silencing mechanism (Rosas-Hernández et al., 2008). Our ChIP-seq analysis revealed that Mss11 not only binds to the upstream regions of *EPA1* and *EPA6* but also associates with the promoters of subtelomeric silencing–related genes, including *SIR4*, *RIF1*, and *RAP1*. Considering the regulatory characteristics of the Epa-encoding genes mentioned above and the confirmed changes in *SIR4*, *RIF1*, *RAP1* expression through RT-qPCR, we hypothesized that Mss11 regulates *EPA1* and *EPA6* expression through both promoter-specific and subtelomeric silencing pathways. However, further researches using luciferase reporter gene assays and gene knockout techniques validating the regulatory pathways of Mss11 on *EPA1* and *EPA6* are warranted.

In summary, this study investigated the functional role of the transcription factor Mss11 in *C. glabrata*. *MSS11* loss was noted to lead to a notable decrease in the CSH, adhesive capability, and biofilm-forming capacity of *C. glabrata*. Furthermore, the *Δmss11* mutant exhibited attenuated virulence in *G. mellonella*. Further investigations revealed that Mss11 regulates *EPA1* and *EPA6* expression, potentially through promoter-specific and subtelomeric silencing pathways. Our results fill the knowledge gap regarding the roles of Mss11 in *C. glabrata*, providing deeper insight into the molecular mechanisms underlying the virulence of the pathogen.

## Data availability statement

The datasets presented in this study can be found in online repositories. The names of the repository/repositories and accession number(s) can be found in the article/**Supplementary Material**.

## Ethics statement

Ethical approval was not required for the studies on humans in accordance with the local legislation and institutional requirements because only commercially available established cell lines were used. Ethical approval was not required for the studies on animals in accordance with the local legislation and institutional requirements because only commercially available established cell lines were used.

## Author contributions

L-LW: Conceptualization, Data curation, Formal analysis, Investigation, Methodology, Visualization, Writing – original draft. S-JH: Data curation, Formal analysis, Investigation, Methodology, Writing – review & editing. J-TZ: Formal analysis, Investigation, Methodology, Writing – review & editing. J-YL: Supervision, Writing – review & editing. M-JX: Conceptualization, Funding acquisition, Supervision, Writing – review & editing.
