# Supplementary material for: Regulatory role of Mss11 in Candida glabrata virulence: adhesion and biofilm formation
Source: Front Cell Infect Microbiol. 2024 Jan 4;13:1321094. doi: 10.3389/fcimb.2023.1321094 (PMC10794409; doi:10.3389/fcimb.2023.1321094)
Supplement: Supplementary file 1 [file Table_1.docx]

Supplementary Material

**Supplementary Table 1 |** MIC50 of different strains in RPMI1640 broth.

|  | MIC50 of strains at 24 h in RPMI1640 broth (µg/mL) | | |
| --- | --- | --- | --- |
| Drug | ATCC 2001 | *Δmss11* | *Δmss11*+*MSS11* |
| fluconazole | 4 | 4 | 4 |
| itraconazole | 0.25 | 0.25 | 0.25 |
| voriconazole | 0.125 | 0.125 | 0.125 |
| caspofungin | 0.125 | 0.125 | 0.125 |
| amphotericin B | 2 | 2 | 2 |
